# What conditions enable decentralization to improve the health system? Qualitative analysis of perspectives on decision space after 25 years of devolution in the Philippines

**DOI:** 10.1371/journal.pone.0206809

**Published:** 2018-11-05

**Authors:** Harvy Joy Liwanag, Kaspar Wyss

**Affiliations:** 1 Swiss Tropical and Public Health Institute, Basel, Switzerland; 2 University of Basel, Basel, Switzerland; 3 Ateneo School of Medicine and Public Health, Ateneo de Manila University, Metro Manila, Philippines; Ministry of Health, KENYA

## Abstract

**Background:**

Decentralization is promoted as a strategy to improve health system performance by bringing decision-making closer to service delivery. Some studies have investigated if decentralization actually improves the health system. However, few have explored the conditions that enable it to be effective. To determine these conditions, we have analyzed the perspectives of decision-makers in the Philippines where devolution, one form of decentralization, was introduced 25 years ago.

**Methods:**

Drawing from the “decision space” approach, we interviewed 27 decision-makers with an average of 23.6 years of working across different levels of the Philippine government health sector and representing various local settings. Qualitative analysis followed the “Framework Method.” Conditions that either enable or hinder the effectiveness of decentralization were identified by exploring decision-making in five health sector functions.

**Results:**

These conditions include: for planning, having a multi-stakeholder approach and monitoring implementation; for financing and budget allocation, capacities to raise revenues at local levels and pooling of funds at central level; for resource management, having a central level capable of augmenting resource needs at local levels and a good working relationship between the local health officer and the elected local official; for program implementation and service delivery, promoting innovation at local levels while maintaining fidelity to national objectives; and for monitoring and data management, a central level capable of ensuring that data collection from local levels is performed in a timely and accurate manner.

**Conclusions:**

The Philippine experience suggests that decentralization is a long and complex journey and not an automatic solution for enhancing service delivery. The role of the central decision-maker (e.g. Ministry of Health) remains important to assist local levels unable to perform their functions well. It is policy-relevant to analyze the conditions that make decentralization work and the optimal combination of decentralized and centralized functions that enhance the health system.

## Introduction

Decentralization is a complex process, but it can be described as the transfer of power or authority over decision-making from higher (e.g. central, federal, or national) to lower levels (e.g. state, regional, cantonal, district, provincial, municipal, or local) of administration [1–3]. It has been emphasized in many countries typically with an overall aim to improve health system performance. De-concentration, devolution, delegation, and privatization are attempts for a typology of decentralization [1,2], although in practice their boundaries overlap rather than clearly distinguish these from one another. This paper focuses on devolution, a type of decentralization where decision-making authority for health services is transferred to lower political levels, often local governments that are largely independent from the higher level of government [1–4].

The arguments in favor of more decentralization in the health sector include: empowerment of local authorities to make decisions on their own; reducing levels of bureaucracy to achieve efficiency; better matching of health services with local priorities; promoting innovations in service delivery that address local needs; and enhancing stakeholder participation in decision-making [3–5]. On the basis of these expected benefits, decentralization has been vigorously promoted in many countries in the last three decades [5].

Has decentralization been effective in achieving the desired reforms? The answer depends on the context, the specific form of decentralization implemented, the health sector functions decentralized, and the outcomes measured. Unlike a concrete intervention, decentralization is rather a process where a standard form does not exist. Given such heterogeneity and a lack of consensus on outcomes for measuring success, any assessment of its effectiveness in improving the health system is challenging. Nevertheless, we have previously argued that these limitations should not be an excuse to abandon the need to assess its effectiveness, given that decentralization continues to be viewed as a strategy for health sector reform [6].

Some of the broader systematic reviews on decentralization of governance of health services have explored: its effects in low and middle-income countries (LMICs) [7] based on the framework of the six building blocks of health systems [8]; the achievements, challenges, and issues related to implementing it in Sub-Saharan African countries [9]; and its impacts on health-related equity [10]. These reviews report both positive and negative outcomes and suggest the consideration of other factors required for successful implementation, such as adequate skills for the local levels taking on the functions [7], political will in the central level to implement changes [9], and the pre-existing socio-economic context within which decentralization is placed [10]. On the other hand, other systematic reviews have examined the effectiveness of decentralizing health service delivery, such as in treatment of MDR-TB patients where treatment success was higher [11], or in providing anti-retroviral therapy for HIV patients where loss to follow-up was less [12]. Both papers however recommend further studies to explore the effectiveness of decentralizing treatment in a range of other settings.

### Decision space approach

One framework for analyzing decentralization is the “decision space” approach [13], which enables analysis of the amount of choice (i.e. wide, moderate, or narrow) transferred from higher to lower levels, the decisions made at local levels within this granted “space,” and the effects that these decisions have on the health system. Previously, decision space has been reported as mostly wide in the Philippines [14], mostly narrow in Ghana [14] and India [15], almost none in Fiji [16], and moderate or varying in Zambia [14], Uganda [14,17], and Pakistan [18,19]. The studies in Pakistan have suggested that a wide decision space at local levels may not be enough to improve service delivery unless it is accompanied by building the capacity of decision-makers who assume the new tasks and by ensuring accountability for the decisions they make. It is therefore useful for policy to explore effectiveness given multiple configurations of decision space with these dimensions of capacity and accountability [20]. We will contribute to this endeavor by an examination of health sector devolution in the Philippines.

### Devolution in the Philippines

**Fig 1:**
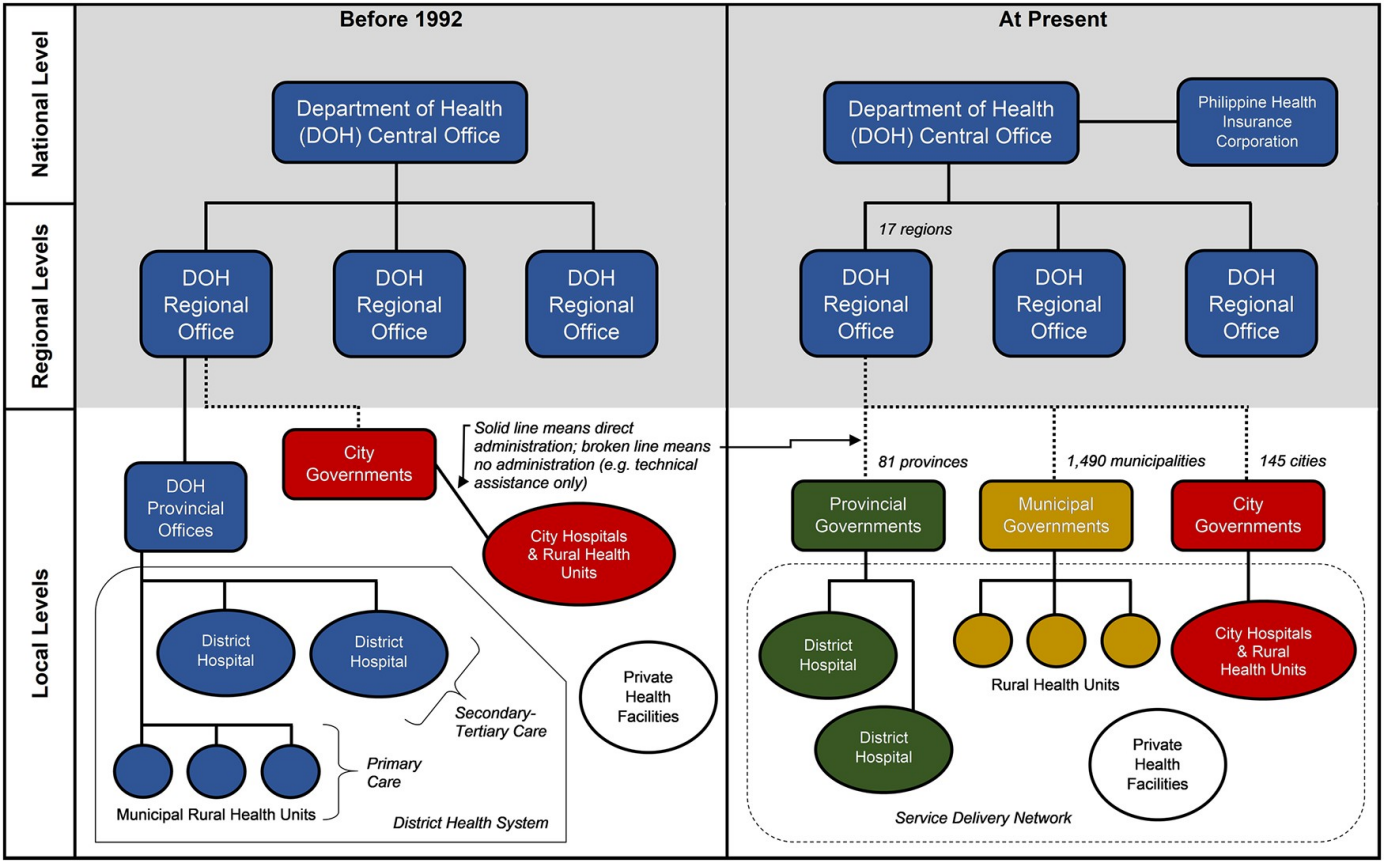
Simplified overview of the administrative structure of government health facilities in the Philippines before and after devolution.

The Philippines is a republic in Southeast Asia comprising >7,600 islands and with a population of 101 million [21]. Government was historically highly-centralized through successive occupations by Spain, the United States, and Japan, followed by independence in 1946, and the dictatorship of Ferdinand Marcos that ended after a peaceful revolution in 1986. Since the 1950s, various waves of decentralization have taken place to disperse the concentration of power in Manila [22,23]. The largest wave culminated in the Local Government Code of 1991 [24] which introduced devolution to the entire archipelago in 1992, at that time considered as the most extensive decentralization in Asia [25]. With assistance from multi-lateral development organizations such as the World Bank, WHO, and USAID [22], the national government transferred the responsibility for government health services and other non-health services (e.g. agriculture) to Local Government Units (LGUs) across the archipelago [22–30]. Currently, the Philippines is organized into 17 regions for the purpose of coordination, although real political power at local levels lies with the LGUs that number into 81 provinces, and the 1,490 municipalities and 145 cities that are geographically within these provinces. Provincial governments maintain some oversight over municipalities and less-urbanized cities, while highly-urbanized cities are completely independent. Devolution disrupted the administrative structure of government health services from what used to be a service under a singular national ministry called the Department of Health (DOH), which previously managed an intact district health system at local levels, into a fragmented service under the control of individual LGUs: provinces responsible for hospitals; municipalities responsible for primary care facilities called Rural Health Units (RHUs); and cities responsible for both levels of care (Fig 1). Concepts such as “Interlocal Health Zones” [23] and “Service Delivery Networks” have been introduced by the national government to restore interlinking by fostering a network of health facilities and providers in contiguous areas, despite these facilities being under different LGUs, to offer a package of health services in an integrated and coordinated manner.

In the peer-reviewed literature, studies reported how devolution in the Philippines failed to enhance community participation in some municipalities [25] and sustained corruption when politicians became the center of decision-making [22] due to what has been described as “elite capture” [31] wherein existing power structures persist despite decentralization, compounded by a lack of accountability measures. In some provinces, inefficiency emerged as a problem when these took on more hospitals than what provincial resources could handle [22,26,28]. In some municipalities, patients learned to cross borders in search for better care [26], while a quality assurance program launched by the national government in 1998 failed to improve quality in primary care centers owned by the municipalities [32]. In the aspect of financing, municipalities, unlike the wealthier cities, continued to rely on the income from the national government for health spending [26]. Moreover, the lack of readiness at local levels prompted the national government to provide a training program in management for local decision-makers [30], and to deploy centrally-hired health professionals to municipalities that have no resources to hire them [33]. One paper on the malaria control program described poor implementation at local levels due to dysfunctional linking with the national level [27].

Consequently, we should then ask: What conditions enable decentralization to produce well-functioning health systems? [6]. We have explored this question by analyzing the perspectives of decision-makers at different levels of the Philippine health system. This is timely not only because of the 25 years of experience of implementing devolution in the Philippines, but also because of current initiatives in the country to change the structure of government from a republican into a federal state [34], indeed a step even further than devolution that will significantly alter how health services will be governed in the country. Lessons from the Philippines can offer policy-relevant insights [29] for countries that have decentralized or are contemplating to adopt some form of decentralization for their health systems.

## Methods

### Semi-structured questionnaire

A semi-structured questionnaire (S1 File) was developed by drawing from the decision space approach and the concept of health sector functions [13], as well as from two studies in Pakistan that analyzed the synergies between decision space, capacity, and accountability [18,19]. The questionnaire provided latitude in exploring participants’ insights and probed their perspectives on and personal experiences in implementing devolution. We examined their flexibility in making decisions within selected health sector functions. These functions were broad categories of tasks where decision-makers make choices for the health sector as previously reported in the studies by Bossert [13,14,18,19]. Drawing from these studies, we initially identified these functions as: 1) planning; 2) health budgeting and financing; 3) human resources for health management; and 4) service delivery.

### Participant selection

We purposively-selected and contacted (via phone calls and emails) decision-makers who were serving the government health sector in positions of authority. Broadly, they represented three groups of decision-makers: 1) ministers and directors from the DOH who served at national and regional levels; 2) provincial, city, and municipal health officers, or those who served as career health officers at local levels; and 3) provincial governors, city mayors, and municipal mayors, or politicians who were elected to head the LGUs at local levels.

### Data collection

The questionnaire was pilot-tested with two potential participants to check for clarity of the questions prior to use. One of the authors (HJL) with training in qualitative research conducted interviews face-to-face with each participant in his/her preferred venue in the Philippines between January and April 2017. HJL is also a Filipino citizen who is familiar with the country’s health system mostly through his work as an academic researcher and who was not employed in the government health service sector. Each interview was audio-recorded and manually transcribed in English. Transcripts were reviewed at least twice to ensure accuracy and subsequently loaded into MAXQDA Standard 12 (VERBI GmbH Berlin, 1995–2017) for coding and analysis.

### Framework method

Analysis was based on the “Framework Method” as previously described in three papers [35–37]. It is considered a systematic approach to thematic analysis that compares and contrasts perspectives. Our approach to analysis combined both deductive and inductive approaches and is summarized as follows: 1) constant familiarization with the data through repeated listening to the audio-recordings while simultaneously reading the transcripts; 2) open coding of the transcripts that identified a preliminary set of categories based on the decision space and the health sector functions; 3) development of an initial analytical framework comprised of these codes and categories being identified from the transcripts; 4) coding of the rest of the transcripts using this analytical framework with continuing iteration whenever new categories were identified; and 5) analysis through comparison of emerging themes across categories, individual interviews, and groups of decision-makers with the use of tables.

Final thematic analysis focused on interpreting: 1) how decision space was exercised by the decision-makers in various health sector functions; 2) whether decision space was seen as wide, moderate, or narrow within each health sector function; and 3) the conditions that make decentralization effective for the health sector in the performance of these functions. We defined a condition as any factor or process (including any potential interaction between these) that has an enabling role in achieving a well-functioning decentralized or devolved health system. Similarly, we also identified those conditions that work in the opposite (i.e. hindering condition). We then summarized these enabling and hindering conditions in a table organized according to health sector functions, together with the decision space within these functions (using blue color coding) across groups of decision-makers. Finally, through an iterative process we synthesized the content of this table into a conceptual diagram, which was inspired by the image of decentralization and centralization previously described in the literature as movements between two opposite poles [2].

### Ethics statement

Written informed consent was obtained from all participants prior to the conduct of the interviews. The study protocol was reviewed and approved in Switzerland by the Ethikkommission Nordwest- und Zentralschweiz (no. 2016-00738) and in the Philippines by the National Ethics Committee (no. 2016-013). Drafting of this paper was guided by the Consolidated Criteria for Reporting Qualitative Research (COREQ) [38].

## Results

### Profiles of the decision-makers

**Fig 2:**
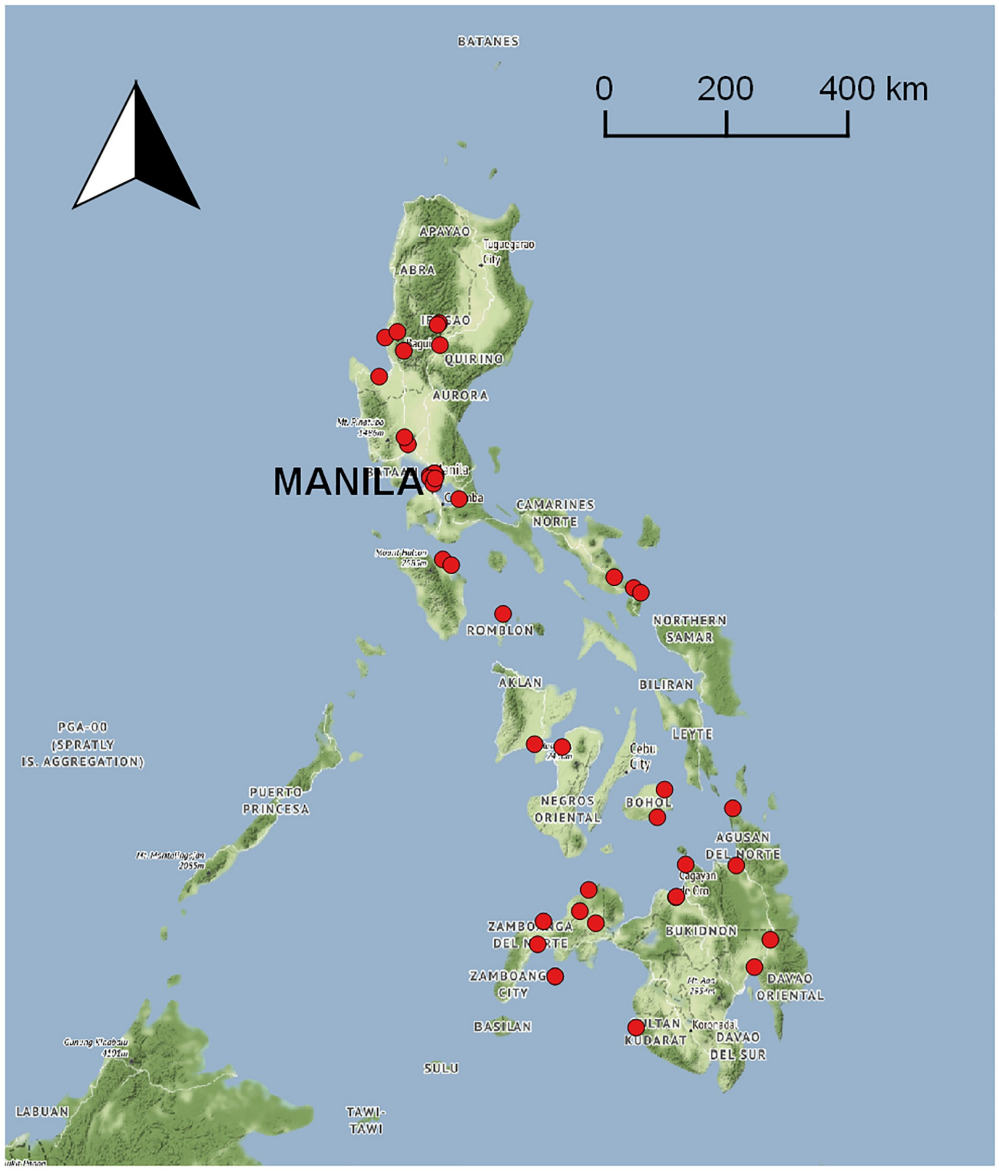
Present and previous areas of health sector-related work of the 27 decision-makers. **Locations indicate assignments that were ≥3 years**. (Map tiles by Stamen Design, under CC BY 3.0. Data by OpenStreetMap, under ODbL.).

We contacted 33 potential participants and interviewed up to 29 decision-makers when saturation was assessed to have already been achieved [39]. The audio files of two interviews were corrupted and subsequently excluded, which nevertheless did not change our judgment of saturation, resulting in a total of 27 interviews transcribed. Each interview lasted an average of one hour and four minutes. The 27 decision-makers worked in a wide range of local settings in the Philippines (Fig 2).

**Fig 3:**
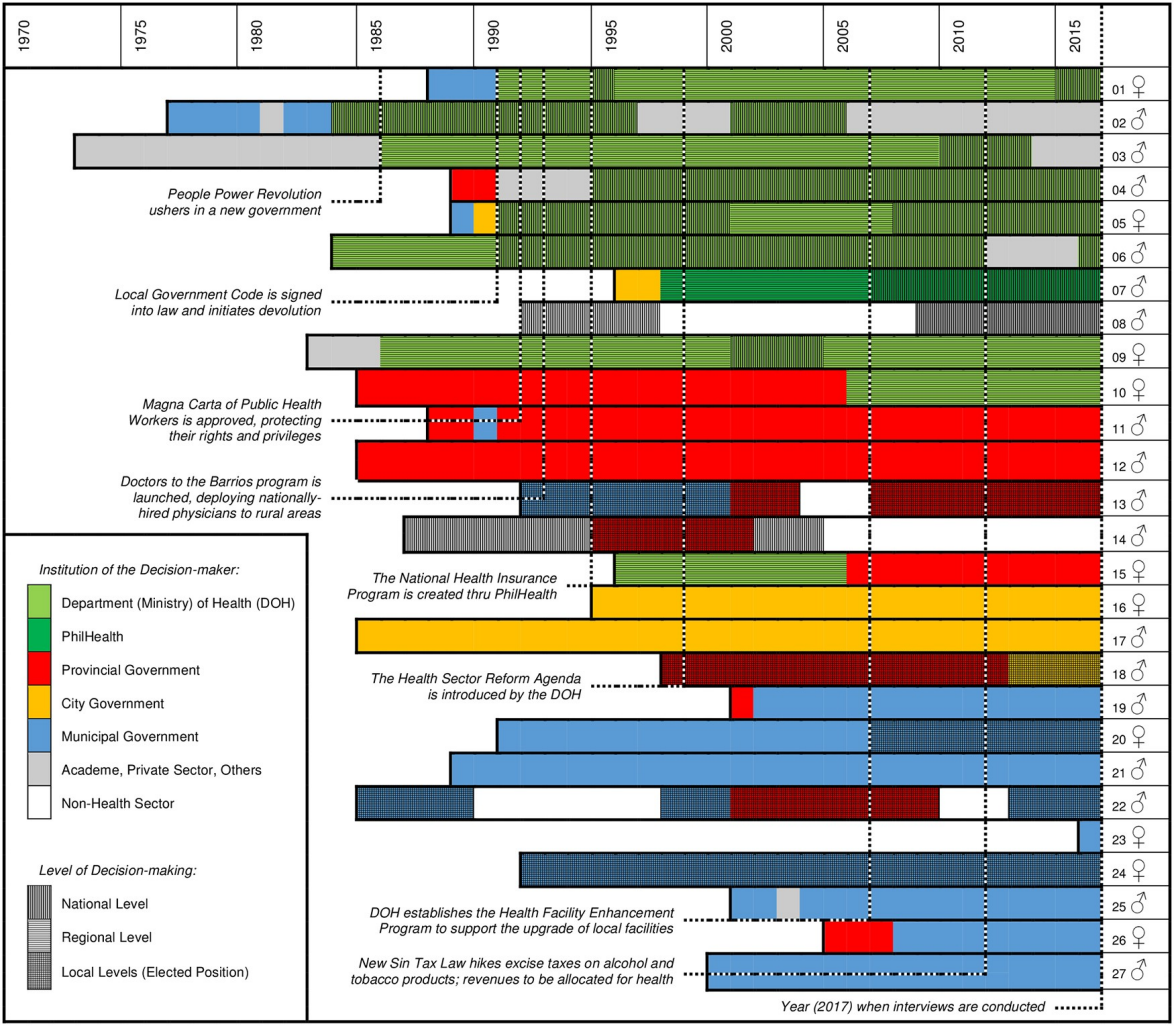
Durations of government service of the 27 decision-makers, the institutions they worked in, and their levels of decision-making. Selected events in the Philippine health sector are also indicated.

There were 17 (63%) males and 10 (37%) females, with an average of 23.6 years of working in the Philippine government sector. At the time of the interviews, 10 (37%) were serving at national and regional levels, 11 (41%) were career health officers at local levels, and six (22%) were elected local officials. Many of them crossed different levels of government during the span of their careers. For instance, nine served in the DOH in various capacities, three of whom were once the Philippine Secretary of Health (i.e. Minister of Health). Among career health officers at local levels were four provincial health officers, three city health officers, and eight municipal health officers, four of whom were heads of their respective national associations of health officers. Among elected officials were three provincial governors, four municipal mayors, one city mayor, two congressmen, and one senator. We further characterized the length of service of each of the 27 decision-makers in Fig 3.

### Health sector functions

The various decision-making activities described by the participants during the interviews led to an expansion of the initial list of health sector functions into five categories, namely: 1) planning; 2) financing and budget allocation; 3) resource management (further divided into “facilities, equipment, and supplies” and “human resources for health” or HRH); 4) program implementation and service delivery; and 5) monitoring and data management.

### Planning

Devolution empowered LGUs to create the Local Health Board (LHB), a multi-stakeholder board chaired by the governor (in provinces) or mayor (in municipalities and cities) that serves as a venue for discussing local health concerns [25]. To what extent the LHB contributes to planning depends on whether it actually meets regularly, as the governor/mayor may choose not to convene it at all, and the ability of members to advocate for the concerns of the sectors they represent. After about 10 years since the introduction of devolution, the DOH instituted the annual “Investment Plan for Health” (IPH) [40] to assist the LGUs in planning and to restore some form of standard planning process. The IPH enables the DOH, through its regional offices, to train the LGUs to develop their annual plans for health, which specify local health needs and the resources from local and central levels to support these needs. Thus, the DOH has become actively involved in planning for local health services and is seen to have a wide (dark blue) decision space in this function compared to local decision-makers whose space may be described as moderate (blue) (Table 1). Conditions that enable decentralization in planning to be more effective for the health system include a functional LHB that feeds into the planning process, as well as opportunities for key decision-makers from central and local levels to meet, negotiate, set priorities, and co-create the local plans together. On the other hand, hindering conditions include a weak mechanism to monitor faithful execution of these plans, and the lack of sustainability for these plans given the reality of elections in the Philippines where local elected officials may change every three years.

**Table 1:** Decision space at central and local levels for the functions of planning and financing and budget allocation (dark blue: Wide decision space; blue: Moderate; light blue: Narrow). Enabling and hindering conditions are described.

Health sector functions	Decision space		Conditions	
Selected questions: *Are you able to…*	Central/Regional decision-makers	Local decision-makers	Enabling	Hindering
**Planning** *Develop your own annual plans for health services at local levels*? *Involve stakeholders in the planning process*? *Implement what has been stated in these plans*?	The DOH sets the national objectives for health, provides the templates for the annual plans and organizes workshops to train the LGUs in preparing their “Investment Plan for Health” (IPH), which will indicate the: 1) local needs to be prioritized; and 2) resources (from central, local, or other sources) to support these needs. Although not legally-bound to submit an IPH, LGUs often participate in the IPH to benefit from the process.	Health officer: He/She prepares the IPH by relying on technical assistance from the DOH. Ideally, the content of the IPH should reflect the articulations of the “Local Health Board” (LHB), composed of stakeholder representatives who meet regularly to discuss health concerns in the locality.	A functional LHB that meets regularly, and where stakeholders actively advocate on behalf of the sectors they represent 1) DOH staff at regional levels who are capable of influencing the LGUs to plan well; 2) local health officer who is skilled in strategic planning and able to work well with his/her elected official; 3) governor/mayor who is supportive of the plans; and 4) an opportunity for these decision-makers to meet, perform priority setting together, and co-create the plans	Weak monitoring of the implementation of plans Lack of an accountability mechanism to incentivize execution of plans and penalize failure of implementation Lack of sustainability of plans as local elected officials may change every three years when local elections are held (i.e. the new governor/mayor who wins may not support continuation of previous plans)
		Elected official: The provincial governor or municipal/city mayor has the authority to convene the LHB and to approve the final version of the IPH. His/her support is essential for the LHB to be functional and for most of the IPH to be implemented.		
**Financing and budget allocation** *Allocate the budget needed to support health services at local levels*? *Create additional sources of financing to support these health services*? *Spend the budget according to what it was intended for*?	Most taxes are collected by the central government, which then allocates the budget at national and local levels. Despite devolution, the DOH share in the government budget has increased substantially in recent years. The allotment that LGUs receive from the central government is often inadequate to support local health services, but the creation of PhilHealth, which administers the national social health insurance program, provided an additional financing mechanism to sustain local health services through reimbursements of services rendered.	Health officer: He/she proposes the annual budget for hospitals or primary care centers which may or may not be approved by the governor/mayor depending on availability of funds. The health officer may also decide on how to spend the additional income from PhilHealth reimbursements, but subject to the guidelines set by PhilHealth.	A high-income LGU (mostly the cities) with several sources of alternative financing (e.g. taxes from local businesses) that are adequate to support local health services A health officer and elected official who are able to work well together and agree on allocating a substantial portion of the local budget for health services A well-funded DOH and PhilHealth that is able to augment the financial inadequacy of low-income LGUs	A governor/mayor (or his/her other subordinates) who interferes in the work of his/her health officer in allocating and spending the budget for local health services, often because of political motivations Concentration of the government budget at central and regional levels without substantially increasing the allotment at local levels, where most government health services have already been devolved
		Elected Official: The governor/mayor has the final decision on how much to allocate in the local budget for health services, which may or may not be increased depending on current resources and priorities. He/she may also interfere in the work of his/her health officer and in the utilization of the additional income from PhilHealth reimbursements.		

The following quote illustrates how planning has provided an opportunity for negotiation between the central and local levels and why it needs to be more strategic:

“*Parts of the plans will be funded by the national government. We work with governors and mayors because the plans emerge from municipal and city levels and integrated at provincial level. The governor presents the consolidated plan and have it approved by the DOH regional office. That’s better because he’s the head of the province and will have ownership of the plan. But sometimes the plan is a wish list, for example, requesting the DOH to finance the fencing of their hospital [laughs]. Planning should be strategic to address real needs and improve their health system*.”

*(Director in the DOH central office*, *28 years in government)*

### Financing and budget allocation

Most financing for health remained within the control of the national government, which pools tax collection and allocates the revenue share of LGUs based on a formula that considers local population and land area. It was the consensus among decision-makers that the inadequate share received by the LGUs led to the chronic underfunding and deterioration of many local health facilities especially in resource-poor provinces and municipalities that have little capacity for locally-generated income. Local health services often competed with other non-health services in budget allocation, which relied on the approval of the governor or mayor. In 1995, the Philippines created the Philippine Health Insurance Corporation (PhilHealth) [41], a DOH-attached agency that manages the national social health insurance program which is financed through premium contributions from enrolled members, most of whom come from the formal sector. Through PhilHealth’s reimbursements for services rendered, many local health facilities received additional financing to sustain operations. In financing and budget allocation, decision space is therefore seen as wide (dark blue) for the central level, and also wide (dark blue) for the local elected official who makes the decision on budget allocations, but moderate (blue) for the local health officer who, in most cases, needs the approval of the elected official when it comes to the local health budget. Enabling conditions include the institutional capacity of LGUs to raise revenues on their own, a well-funded central agency able to augment the lack of financial resources at local levels, and effective collaboration between the local elected official and health officer to be able to agree on allocating a substantial portion of the local budget in favor of health services. Hindering conditions include the concentration of financial resources at central levels despite devolution, and budget utilization that is driven mostly by political motivations [42] instead of genuine health needs.

The following quote illustrates an example of how central support helps the LGUs meet their needs in terms of financing and budget allocation:

“*I kept talking to the municipalities to fix their RHUs. Of course, they have their allotment from taxes collected by the national government. But the important thing is for LGUs to understand that their operations are sustainable. How? They spend PHP100,000 (USD2,000) from their own budget to upgrade the RHU and have it accredited by PhilHealth as a maternal delivery unit. PhilHealth will pay PHP8,000 (USD160) for every delivery. How many deliveries, 30 per month? They get back PHP240,000 (USD4,800) per month. And that’s just for maternal health. The RHUs do many other things that PhilHealth will pay for*.”

*(Former Philippine secretary of health or* “*minister of health*”*)*

### Resource management

Despite devolution, the DOH continued to purchase the supplies needed for most public health programs, and these supplies are given to the LGUs as augmentation for their health facilities. In 2007, the DOH also initiated the “Health Facilities Enhancement Program” (HFEP) which provided a mechanism for LGUs to request assistance in the construction or upgrade of health facilities through funds from the national government. The DOH also established a national rural physician deployment program called “Doctors to the Barrios” [33] one year after the introduction of devolution which enabled the national government to hire physicians who are then deployed as local health officers in resource-poor municipalities that lack them. Deployment has since expanded to include nurses, midwives, medical technologists, and dentists. Under this program, deployed HRH receive their salaries from the national government but perform their duties as local HRH serving the LGUs. In some LGUs that have adequate resources to hire their own HRH, the governor or mayor has the supervisory authority over local HRH. Therefore, decision space for resource management overall is seen as wide (dark blue) for the central level, while at local levels it is moderate (blue) in terms of managing facilities, equipment, and supplies. However, for HRH management at local levels, decision space is seen as narrow (light blue) for the local health officer but wide (dark blue) for the local elected official who, in practice, is in full control of the hiring and firing process (Table 2).

**Table 2:** Decision space at central and local levels for the functions of resource management, further classified into facilities, equipment, and supplies and human resources for health (dark blue: Wide decision space; blue: Moderate; light blue: Narrow). Enabling and hindering conditions are described.

Health sector functions	Decision space		Conditions	
Selected questions: *Are you able to…*	Central/Regional decision-makers	Local decision-makers	Enabling	Hindering
Resource management				
**Facilities, equipment, and supplies** *Put up the appropriate types of health facilities in the areas where these are needed*? *Maintain and upgrade these facilities*? *Provide adequate equipment and supplies*, *including medicines*, *for these facilities to meet the needs of the population you serve*?	The DOH maintains tertiary care hospitals in every region and highly-specialized hospitals in the capital where patients from local health facilities can be referred for further management. The DOH and PhilHealth also have the regulatory power of licensing and accreditation, respectively, which ensures quality in health facilities. In 2007, the DOH established the “Health Facilities Enhancement Program” (HFEP) where resources from central levels are channeled towards the construction or upgrade of local health facilities (including equipment) owned by the LGUs. The DOH also continues to purchase supplies for many public health programs (e.g. vaccines, TB drugs, iron supplements for pregnant women, contraceptives, etc.). PhilHealth has also provided guidelines instructing LGUs to spend their PhilHealth income only for health-related expenses.	Health officer: He/She manages the hospitals (in provinces and cities) or the RHUs (in municipalities and cities). However, his/her success in maintaining these facilities relies largely on the budget approved by the governor/mayor. The HFEP provides an opportunity to address this gap.	A health officer (a physician as prescribed by the law) who has adequate skills for effectively managing health facilities and programs and is innovative in finding ways to improve service provision (e.g. public-private partnerships for service delivery) A governor/mayor who sees the hospital or RHU as an important component of his/her term of office that affects his/her chances of re-election A well-funded DOH and PhilHealth able to augment the needs for facilities, equipment, and supplies by the LGUs, as well as the additional compensation needed for local HRH	Loss of leverage in bulk procurement as LGUs have to negotiate individually with suppliers to procure equipment and supplies potentially at higher prices Less autonomy for some local hospitals after these were transferred to LGUs, and hospital administrative matters combined with other non-health services which all go through the bureaucracy in provincial governments (leading to reduced efficiency) In some cases, poor coordination between the DOH and the LGUs in the provision of augmentation that may result in construction of incomplete health facilities, or facilities that have a faulty design, or equipment/medicines delivered to LGUs that do not match what is actually needed
		Elected official: The quality of local health facilities often reflects how much the governor/mayor prioritizes health. For example, the governor may view provincial and district hospitals as an unnecessary burden that provincial resources could not maintain and thus should be returned to the management of the DOH.		
**Human resources for health (HRH)** *Hire (or fire) the appropriate types and number of HRH which your local population requires*? *Compensate HRH commensurate to their workload and according to national standard rates*? *Build the capacity of these personnel and support their career development*?	The DOH established deployment programs where the national government hires physicians, nurses, midwives, dentists, and medical technologists who are deployed to serve in local health facilities owned by LGUs that lack the capacity to hire them. The DOH is also a major capacity building provider for local health officers who are invited to participate in regular training activities for implementing public health programs. PhilHealth has also required that a portion of its reimbursements to LGUs be used as additional compensation for local HRH.	Health officer: Despite a law that standardized the salaries and benefits for HRH, some local health officers receive a lower compensation compared to others due to the lack of funds available for salaries especially in resource-poor LGUs. The differences in compensation has been identified as a cause of low morale among affected health officers. In some cases, health officers may also be unjustly sidelined or placed on probation by a newly-elected governor/mayor who wishes to place somebody else in the position.	Local health officers who are non-partisan during local elections and, thus, insulate themselves from possible political harassment whenever there is a change in the governor/mayor Strongly-united associations of local health officers that have the leverage to engage the DOH, PhilHealth, and elected local officials to assert their rights and privileges A governor/mayor who values the important role played by HRH and thus promotes their rights and privileges Adequate capacities at central level to hire additional HRH to be deployed to meet the needs at local levels, and also to augment the compensation of local HRH already hired by LGUs unable to provide their full salaries	Inclusion of local health services, which is labor-intensive, into auditing regulations that limit hiring of personnel Weak accountability for LGUs that do not provide the full compensation and benefits that local HRH legally deserve Lack of a seamless career stepladder for local health officers whose careers are mostly confined within the LGUs that hire them (unlike in a centralized system where they may be seamlessly promoted to positions at regional or national levels) In some cases, tension between the DOH and the LGUs for control over health officers who are invited to participate in capacity building initiatives provided by the DOH but who are administratively under the LGUs that control their ability to participate
		Elected official: The governor/mayor makes the decision in hiring and firing. In some cases, hiring is based not on qualifications but on political patronage. Moreover, hiring of additional HRH to meet the demands of an increasing population is not always possible because of a limit imposed by the government’s auditing body on the proportion of the local budget that can be used for salaries. This cap has resulted in the hiring of many contractual HRH without security of tenure.		

The following quote illustrates an example of how the devolution of HRH management led to inadequate compensation for local HRH especially in resource-poor LGUs:

“*Public health workers are at the mercy of the LGUs in terms of salaries and benefits. The compensation enacted by the national government should also be given to local health workers. But the implementation of the standard salary rates is not the same across the country because the LGUs always say that they are autonomous from the national government. So the health workers in municipalities, cities, and provinces where the benefits are being given are lucky. But the others who don’t get these benefits still need to lobby for their rights*.”

*(Municipal health officer in a low-income island*, *16 years in government)*

On the other hand, the following quote illustrates the continuing significant intervention of the central government in providing for the various resources needed by the LGUs for better service delivery at local levels:

“*There is creeping re-centralization in infrastructure, equipment, and human resource. The DOH also procures all commodities for most of the major public health programs. TB drugs and vaccines are entirely procured by the DOH and given to the LGUs, and the LGUs no longer need to buy anything. What else is devolved there? If you would look at the Philippine national health accounts, LGU expenditures for health are going down while the budget of the DOH is getting higher*.”

*(Philippine undersecretary of health or* “*deputy minister*,” *28 years in government)*

Some of the enabling conditions include: a governor or mayor who considers local health services as an important component of his/her administration and is supportive of the needs of local HRH; a local health officer who has good management skills, refrains from partisan politics, and is actively involved in the association of health officers who are able to use their influence as a group to assert their rights and privileges; and a DOH and PhilHealth with adequate resources to augment resource needs at local levels. On the other hand, one hindering condition, particularly in areas that host deployed HRH as augmentation for their lack of staff, is the potential tension between the local mayor, who is the head of the LGU, and the deployed HRH, who is technically an employee of the DOH. In such a situation, conflict sometimes arises because of the ambivalence in the lines of authority when the agency responsible for managing the decentralized service is different from the agency providing the salary of the staff tasked to deliver that service. Other hindering conditions include: weakened leverage in negotiating prices of supplies and equipment when devolution obliged LGUs to negotiate individually with suppliers in procuring what is needed at local levels; weak accountability for LGUs when these do not provide the full range of salaries and benefits that local HRH legally deserve; and the lack of a stepladder for local health officers to pursue their career aspirations as the devolved structure limits their opportunities for promotion within the LGU where they are employed.

### Program implementation and service delivery

Devolution provided opportunities for LGUs to develop and implement local health programs that address their own unique needs, especially in settings with a culturally-sensitive context or where access to care is geographically-challenging. However, most LGUs still continued to rely on the DOH for technical assistance in the implementation of many public health programs (e.g. Expanded Program on Immunization, Family Planning Program, TB Control Program, Environmental Health Program, etc.), which are determined and planned at the national or central level and cascaded down through the DOH regional offices for implementation by the LGUs at local levels. Moreover, health facilities located in the same area may have limited means of effective cooperation between one another when these facilities are owned by different LGUs and only artificially linked through informal networks. In this context, decision space is viewed as wide (dark blue) for the central level and moderate (blue) at local levels for both the health officer and the elected official (Table 3).

**Table 3:** Decision space at central and local levels for the functions of program implementation and service delivery and monitoring and data management (dark blue: Wide decision space; blue: Moderate; light blue: Narrow). Enabling and hindering conditions are described.

Health sector functions	Decision space		Conditions	
Selected questions: *Are you able to…*	Central/Regional decision-makers	Local decision-makers	Enabling	Hindering
**Program implementation and service delivery** *Implement health programs that are mandated by the national government*? *Provide your own unique health programs or services that address local priorities and consider the local context*? *Provide local health services that meet the standards for quality*?	The DOH sets the national policies, technical guidelines, and standards for service delivery. For example, the overall strategic plans for many disease control programs (e.g. TB, malaria, non-communicable diseases, etc.) are determined by the DOH at the central level and cascaded down to the LGUs through its regional offices. Most of the health programs implemented at local levels are DOH-determined programs.	Health officer: Depending on his/her capacity for innovation, the health officer may conceptualize and implement unique programs that address local health needs.	Opportunities for innovation in service delivery that consider, for instance, the cultural sensitivities of particular communities, or the challenging landscape that affects access to care Strong leadership by the DOH to provide technical assistance to the LGUs for implementing national public health programs and in dealing with health issues that are beyond the capacity of these LGUs (e.g. protocols during outbreaks or health emergencies, guidelines for introducing a new vaccine, etc.)	Weak mechanism for ensuring that program implementation at local levels is faithful to the standards set at the central level Weak interlinking for resource-sharing and seamless patient referrals between local health facilities owned by different LGUs but located in the same catchment area
		Elected official: Depending on his/her interest in health, the governor/mayor may or may not be actively-involved in the implementation of health programs. Nevertheless, his/her support is critical for successful implementation of any program.		
**Monitoring and data management** *Choose the indicators for monitoring the performance of the health system at local levels*? *Collect these indicators in an accurate and timely manner*? *Perform data management efficiently and electronically*?	The DOH monitors a list of indicators through the “Field Health Service Information System” (FHSIS) which is published annually, although often 2–3 years delayed due to the difficulty of completing the data coming from local levels. Efforts have been initiated at central levels to make data management more efficient by making LGUs adopt electronic tools for data collection and submission to the DOH.	Health officer: He/she is responsible for ensuring that all relevant health indicators requested by the DOH are collected by his/her staff and submitted to the DOH, which compiles the data. There is, however, no strict penalty for late submission of reports, or for submission of inaccurate data.	Standardization at central levels of a list of relevant health indicators for strict collection at local levels Availability of electronic tools for performing monitoring and data management more efficiently	Fragmented data monitoring and management system with weak central control for timely collection of accurate data at local levels Use of multiple electronic tools for data collection by different LGUs, resulting in lack of harmonization of data transmission for consolidation at the central level
		Elected official: The governor/mayor is often not involved in monitoring and data management and fully delegates this function to his/her health officer.		

The quote below illustrates an example from one province on how devolution has allowed the LGU to deliver health services that are suitable to the local context:

“*We are indigenous peoples, and we have practices that are culturally-appropriate but may be frowned upon at the national level. While we advocate for facility-based deliveries, in geographically-isolated areas, mothers deliver in the house. When I was mayor, we provided training for the husbands because, in our culture, the person who delivers, aside from the midwife, is the traditional village birth attendant or the husband. So at least there is basic training for the husbands. That was our innovation. We also designed our local hospitals so that there are areas where the patient’s family can stay to have an atmosphere like home*.”

*(Former provincial governor and municipal mayor*, *26 years in government)*

Enabling conditions for decentralization to be effective include opportunities for innovation for local decision-makers to improve service delivery, as well as strong leadership on the part of central decision-makers to provide continuing technical guidance to the local levels for program implementation. Hindering conditions include a weak mechanism to ensure fidelity of program implementation at local levels [27], and weak interlinking between local health facilities owned by different LGUs but nevertheless located in the same catchment area, which has reduced opportunities for resource-sharing and a seamless patient referral scheme.

### Monitoring and data management

The Field Health Services Information System (FHSIS) [43], which is managed by the DOH, contains the official health data of the Philippine government. With devolution, the seamless flow of data from local levels to regional and central levels to complete the FHSIS has become more challenging, especially with the loss of direct administration by the DOH over data reporting by LGUs. Nevertheless, efforts have been initiated to help facilitate data management by promoting the use of different electronic tools for data transmission from local levels. Thus, decision space for monitoring and data management for the central level is seen as moderate (blue), while decision space is wide (dark blue) for the health officer who controls data collection at local levels and narrow (light blue) for the elected official who has little involvement in performing this function.

The following quote is an illustration of how devolution has made it more difficult to harmonize the collection and pooling of health-related data at the national/central level:

“*We try to publish the FHSIS final report every year. We are having a bit of difficulty, especially in some areas, in collecting the data. But with all the support that we are providing to the LGUs, it is easier to make them obey and submit their reports to us. Previously, we tried e-FHSIS and we gave computers to the LGUs, but they were not able to encode the data, and there were problems with connectivity. The final FHSIS report is usually 2–3 years delayed because it takes a long time to collect the data from all these LGUs*.”

*(DOH regional director*, *34 years in government)*

**Fig 4:**
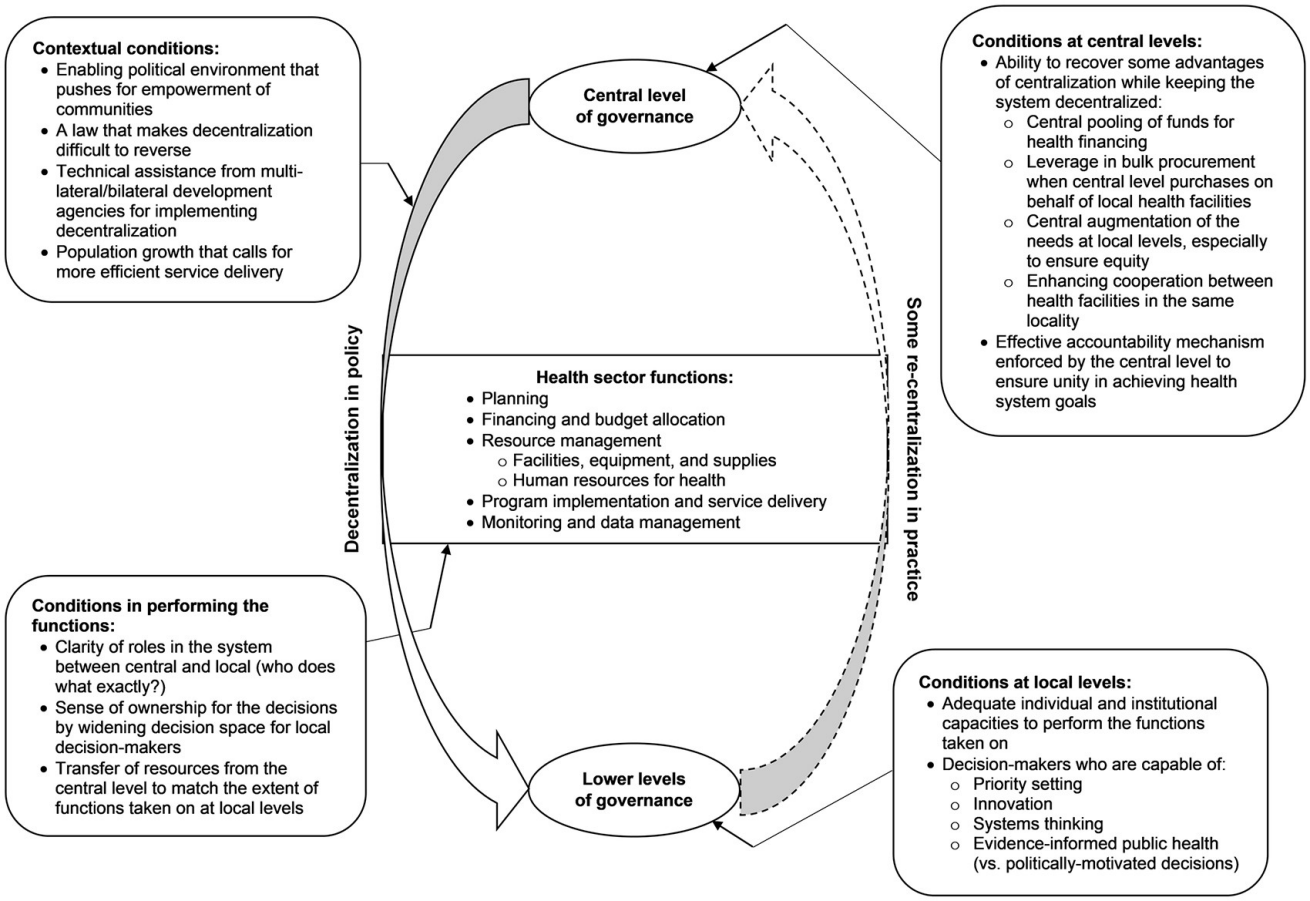
A conceptual diagram inspired by the image of decentralization and centralization as movements between two opposite poles. Various conditions to be considered for decentralization to be effective in improving the health system are proposed.

The conditions that either enable or hinder decentralization to improve the health system are several, and our exploration of decision-making within the five health sector functions provided a more organized way of capturing these conditions. Using the image of decentralization and centralization as a movement between two opposite poles [2], we have further synthesized these conditions in a conceptual diagram that mapped where these conditions should be considered in terms of performing the functions, and in terms of decision-making at central and at local levels (Fig 4). In this figure, we have also included some of the conditions in the broader context where decentralization is placed based on the experience in the Philippines. Some of these contextual conditions include an enabling political environment and a law that makes decentralization difficult to reverse, the supporting role played by multi-lateral/bilateral development organizations that provide technical assistance in implementing devolution, and the increasing population which, particularly in the Philippines, calls for a more efficient delivery of health care at local levels.

Conditions related to the performance of the decentralized functions include clarity of roles for the various decision-makers in the system, a sense of ownership for the decisions they make, and the transfer of sufficient resources to support performance of these functions at local levels. Conditions at local levels include adequate capacities which, at the individual level, should include priority setting, innovation, systems thinking, and evidence-informed public health. Lastly, conditions at central levels include the ability to enforce an effective accountability mechanism, and to recover some of the advantages of centralization, such as in pooling of funds for more efficient financing, gaining leverage through bulk procurement of supplies on behalf of local health facilities nationwide, central augmentation of the needs at local levels especially to ensure equity, and enhancing cooperation between local health facilities in the same locality. The experience of health sector devolution in the Philippines suggests that decentralization can be implemented in policy but, in practice, some forms of re-centralization take place to make up for the inadequacies at local levels that took on the functions. Thus, one of the challenges in devolving the health sector is identifying the right combination of decentralized and centralized functions, even as the health system remains broadly decentralized, in order to achieve optimal health system performance.

## Discussion

This paper aimed to determine the conditions that potentially make decentralization effective in improving the health system by analyzing the experience of devolving government health services in the Philippines. Our analysis of qualitative data has allowed us to explore the variety of factors and processes in the system, which we have called conditions, that play a role in enabling (or hindering) the effectiveness of decentralization. Rather than quantify these conditions through the calculation of composite indices, we have instead shown the feasibility of obtaining a more nuanced and contextualized understanding of decision-making when these conditions play out in particular situations, which also provides specific opportunities for policy interventions. For example, rather than make a general statement that accountability is weak in the health sector function of planning, the qualitative approach has allowed us to explore practical ways to improve decision-making in this function. One concrete policy intervention for the Philippines is the monitoring of the execution of these plans when the central level provides incentives to LGUs for satisfactory accomplishment and imposes penalties for failure in implementation.

The experience of devolution in the Philippines is consistent with the idea that decision space is closely linked to the concept of control. Widening decision space in practice means that control over health services is granted to one group of decision-makers over another. At local levels, decision-making in most functions is concentrated with the elected local official, a politician who may or may not be supportive of public health goals, rather than the local health officer (almost always a physician) who holds the technical and administrative competence for health services. The politicization of health has been blamed by all decision-makers in this study as a hindering condition commonly experienced across most health sector functions, often in the function of managing HRH. How to address an issue as serious as this in the Philippines is not easy as politics is unavoidable in healthcare, although some approaches have been described by the decision-makers themselves that include, for example: building the capacity of the local elected official to understand that health must be a priority; ensuring that the local health officer refrains from partisan local politics; and making the national government (i.e. DOH and PhilHealth) use its leverage over LGUs to promote the rights and privileges of local HRH.

Furthermore, granting the decision space in favor of decision-makers at local levels through decentralization or devolution does not necessarily imply that it is best for the central level to relinquish entirely its control over decision-making. The goal, rather, is to identify the optimal combination of decentralized and centralized functions. Some of the recent studies, such as the one on Fiji [16], have argued that the failure to reap the full benefits of decentralization for the health sector was in part due to the lack of a completely wide decision space at local levels in spite of decentralization in policy. Similar observations on this lack of decision space at local levels despite decentralization has been noted in the management of county health facilities in Kenya [44,45], or the control over HRH by district heath managers in Uganda [17], or most of the health sector functions in selected districts in India [15]. In the case of the Philippines where local decision-makers are ill-prepared or lack the capacity to fulfill their health sector functions well, having some wide decision space for the central decision-maker may actually be a sign that the central level is intervening in ways that assist the local levels. Our analysis indicates that, with the exception of high-income LGUs (e.g. in highly-urbanized cities), many health sector functions in the Philippines are performed by local decision-makers with significant augmentation from the central level, without which the health system would most likely have been in a worse situation. Thus, contemplating decentralization for the health sector in any setting should seriously consider the readiness of the lower levels of administration to assume the new functions, as well as analyze the evolving role that the central level (e.g. Ministry of Health, or DOH and PhilHealth in the Philippines) has to play as it learns to implement decentralization and shepherds the health system as a whole. Certainly, some form of coordination must be maintained at the central level no matter how extensive the form of decentralization [46], and some tradeoffs must be negotiated for clarity of roles among decision-makers at different levels of the health system [47]. The Philippines is an example of how the central level could use its regulatory power and the augmentation it provides as leverage to build capacities at local levels and also make them accountable for their decisions.

## Conclusions

In summary, several conditions that enable or hinder the effectiveness of decentralization for the health sector have been described in this paper by analyzing the perspectives on decision-making in five functions. For planning, these conditions include a multi-stakeholder approach, being strategic, and monitoring execution. For financing and budget allocation, these include capacities to raise revenues for health services at local levels, more evidence-informed and less politically-motivated funding decisions, and effective central pooling of funds for augmenting financing needs at local levels. For resource management, these include having a central level capable of providing resource needs at local levels by using the leverage in bulk procurement and deploying the HRH needed in areas that lack them, as well as having a good working relationship between the local health officer and the elected official. For program implementation and service delivery, these include promoting innovation at local levels while the central level ensures fidelity to national objectives and likewise promotes cooperation among local health facilities. Finally, for monitoring and data management, these include the central level being capable of ensuring that data collection from local levels is performed in a timely and accurate manner despite the system remaining devolved. One important condition is the role maintained by the central decision-maker especially in assisting local levels unable to perform their functions well. It will be useful for policy to explore the optimal balance of decentralized and centralized functions, even as the system remains decentralized overall, and focus on the conditions that have to be in place in order for decentralization to be effective in improving the health system.

The experience of devolution in the Philippines highlights the reality that decentralization is a long and complex journey and not an automatic solution for enhancing health system performance. Particularly for the Philippines, this means that current initiatives to expand decentralization even further by changing the structure of government from a republican into a federal form must be very carefully re-examined, especially in terms of how such a change would, once again, impact the effectiveness of health service delivery at local levels. Our findings also provide an opportunity for comparison with the experience in other countries that have adopted decentralization and assess similarities (or differences) in lessons learned. Any country that contemplates whatever form of decentralization for its health sector must recognize that the presumed benefits do not happen overnight, and that expectations must be tempered by the challenges of implementing it on the ground.

## Supporting information

S1 File Analyzing the effectiveness of decentralization in improving the health sector with a focus on the Philippines. Key Informant Interview (KII) Guide.
(PDF)

## Abbreviations

COREQ: Consolidated Criteria for Reporting Qualitative Research
DOH: Department of Health
FHSIS: Field Health Services Information System
HFEP: Health Facilities Enhancement Program
HIV: Human Immunodeficiency Virus
HRH: Human Resources for Health
IPH: Investment Plan for Health
KII: Key Informant Interview
LGUs: Local Government Units
LHB: Local Health Board
LMICs: Low and Middle-Income Countries
MDR-TB: Multi-Drug Resistant Tuberculosis
PhilHealth: Philippine Health Insurance Corporation
PHP: Philippine Peso
RHUs: Rural Health Units
TB: Tuberculosis
USAID: United States Agency for International Development
USD: United States Dollar
WHO: World Health Organization
