# Supplementary material for: What conditions enable decentralization to improve the health system? Qualitative analysis of perspectives on decision space after 25 years of devolution in the Philippines
Source: PLoS One. 2018 Nov 5;13(11):e0206809. doi: 10.1371/journal.pone.0206809 (PMC6218067; doi:10.1371/journal.pone.0206809)
Supplement: S1 File — Key Informant Interview (KII) Guide. (PDF) [file pone.0206809.s001.pdf]

Swiss TPH

Swiss Tropical and Public Health Institute
Schweizerisches Tropen- und Public Health-Institut
Institut Tropical et de Santé Publique Suisse

Associated Institute of the University of Basel

Analyzing the Effectiveness of Decentralization
in Improving the Health Sector with a Focus on the Philippines

Key Informant Interview (KII) Guide

Introduction

- Introduce yourself
- Say thank you for his/her availability for the interview
- Provide an overview of the research
- Go through the informed consent form (ICF)
- Have the ICF signed; one copy goes to the informant
- Ask the interviewer if he/she has any other questions
- Begin audio recording
- Start the interview

Date Today:	Time started:	Interviewer: **Harvy Liwanag**

**Informant's profile**	Informant's code:

Name:	Sex:	Age:
Institution:	Address:	No. of years working in the public health sector:

Level of involvement in decision-making in the public health sector:

- ☐ National level
  - ☐ *Department of Health* ☐ *Other:*
- ☐ Regional level
  - ☐ *Department of Health* ☐ *Other:*
- ☐ Provincial level
  - ☐ *Governor* ☐ *Health officer* ☐ *Other:*
- ☐ Municipal level
- ☐ City level
  - ☐ *Mayor* ☐ *Health Officer* ☐ *Other:*
- ☐ Other:

**Knowledge on devolution**
1. Would you consider the implementation of devolution of health services as one of the major challenges of the Philippine health sector?<br>☐ Yes → Please explain why.<br>☐ No → Please explain why not, and tell me what you consider instead as a major challenge for the health sector.
2. What does devolution mean to you?
3. In your opinion, what do you think was the rationale behind the decision to implement devolution of health services in the Philippines in 1991?
**Personal experience with devolution (if applicable)**
4. Could you please tell me more about any personal experience during the shift of the governance of health services from a centralized to a devolved arrangement in the early 1990s?
**Perceptions on the impact of devolution**
5. Based on your experience working in the health sector, what were the <u>positive effects</u> of devolution of health services to the Philippine health sector?
6. Based on your experience working in the health sector, what were the <u>negative effects</u> of devolution of health services to the Philippine health sector?
7. In your opinion, <u>overall</u>, did devolution strengthen or weaken the Philippine health system? Please explain why.
**Opinion on the future of devolution**
8. In your opinion, should the current set-up of devolved governance of health services be maintained?<br>☐ Yes → Please explain why.<br>☐ No → Please explain why not and tell me what changes you would recommend.<br>☐ Don't know → Please tell me why.
Now, let me ask questions related to how you make decisions in your work in the public health sector. . .
**Strategic and operational planning**
Decision Space

9. Strategic and operational planning refers to, as the term suggests, future planning for health services at local levels. In your current (or previous) work, could you please tell me more about examples of decisions that you are making within the scope of strategic and operational planning?<br>*Probe: Are you able to*<br>• *Prepare a local plan for health services?*<br>• *Perform priority setting (e.g. determine local priorities that may be different from national priorities?)*<br>• *Undertake monitoring and evaluation that may include indicators different from those set by the national government?*<br>• *Others?*
10. In each of the examples that you described, would you say that you are able to make decisions comfortably well? Or are you having difficulties in making such decisions <u>and</u> in executing those decisions? Please explain further.<br>*Probe: What usually are your considerations (or what do you look at) that lead to your decisions here?*<br>*Probe: Is your actual decision the desired one? Why or why not?*<br>*Probe: Ask informant to classify his/her decision space for this function into none, narrow, moderate, or wide.*<br>*Probe: What changes in decision space are necessary for this function? And what changes in the conditions are needed to effect such changes?*<br>*Probe: How do you assess if the decisions you made were right or wrong?*<br>*Probe: Do you make an attempt to compare or share experiences with other regions/provinces/LGUs etc.?*
Accountability
11. Could you please tell me what <u>accountability</u> means for you?
12. In your opinion, do you think that the decisions you are making related to strategic and operational planning are considered <u>accountable</u>? Please explain further.<br>*Probe: If not accountable, ask informant to elaborate on how to make decision-making more accountable.*
13. Could you please tell me what <u>transparency</u> means for you?
14. In your opinion, do you think that the decisions you are making related to strategic and operational planning are considered <u>transparent</u>? Please explain further.<br>*Probe: Can the public easily access information related to strategic and operational planning?*<br>*Probe: If not transparent, ask informant to elaborate on how to make decision-making more transparent.*
15. Are you able to ensure <u>an inclusive approach</u> to decision-making? How are <u>stakeholders able to participate</u> in strategic and operational planning?

Probe: At the end of the day, is your decision an individual decision or a collective decision?
Probe: If an inclusive approach is not adequate, ask the informant to elaborate on how to make it adequate.

16. In your opinion, who are the (3) three most important actors or stakeholders who should be making decisions regarding strategic and operational planning for health services at local levels? Please explain why.
Probe: Which institution should take the lead in coordinating such decision-making?

Capacity

17. Could you please tell me what capacity means for you?

18. In your personal assessment, would you say that you have an adequate capacity for making decisions related to strategic and operational planning? Please explain further.

19. What do you think could be strategies to build capacity for decision-making in strategic an operational planning for health services in the Philippines?
Probe: Who or which agency should be implementing the capacity building strategies suggested?

Health budgeting or financing

Decision Space

20. Health budgeting or financing refers to decisions on resources to be allocated for health services at local levels, including which funds should come from central sources and which funds should come from local sources. In your current (or previous) work, could you please tell me more about examples of decisions that you are making within the scope of health budgeting or financing?
Probe: Are you able to
    - *Determine the local budget for health?*
    - *Monitor how the local budget for health is being spent?*
    - *Raise local taxes to fund local health services?*
    - *Charge fees for the use of health services?*
    - *Introduce new and innovative ways for local revenue generation?*
    - *Decide how to spend the funds coming from the central government (e.g. PhilHealth, DOH)?*
    - *Others?*

21. In each of the examples that you described, would you say that you are able to make these decisions comfortably well? Or are you having difficulties in making such decisions and in executing those decisions? Please explain further.
Probe: What usually are your considerations (or what do you look at) that lead to your decisions here?
Probe: Is your actual decision the desired one? Why or why not?

Probe: Ask informant to classify his/her decision space for this function into none, narrow, moderate, or wide.<br>*Probe: What changes in decision space, if any, are necessary for this function? And what changes in the conditions are needed to effect such changes?*<br>*Probe: How do you assess if the decisions you made were right or wrong?*<br>*Probe: Do you make an attempt to compare or share experiences with other regions/provinces/LGUs etc.?*
Accountability
22. In your opinion, do you think that the decisions you are making related to health budgeting or financing are considered accountable? Please explain further.<br>*Probe: If not accountable, ask informant to elaborate on how to make decision-making more accountable.*
23. In your opinion, do you think that the decisions you are making related to health budgeting or financing are considered transparent? Please explain further.<br>*Probe: Can the public easily access information related to health budgeting or financing?*<br>*Probe: If not transparent, ask informant to elaborate on how to make decision-making more transparent.*
24. Are you able to ensure an inclusive approach to decision-making? How are stakeholders able to participate in health budgeting or financing?<br>*Probe: At the end of the day, is your decision an individual decision or a collective decision?*<br>*Probe: If an inclusive approach is not adequate, ask the informant to elaborate on how to make it adequate.*
25. In your opinion, who are the (3) three most important actors or stakeholders who should be making decisions regarding health budgeting or financing at local levels? Please explain why.<br>*Probe: Which institution should take the lead in coordinating such decision-making?*
Capacity
26. In your personal assessment, would you say that you have an adequate capacity for making decisions related to health budgeting or financing? Please explain further.
27. What do you think could be strategies to build capacity for decision-making in health budgeting or financing in the Philippines?<br>*Probe: Who or which agency should be implementing the capacity building strategies suggested?*
**Human resources for health (HRH) management**
Decision Space
28. This health sector function refers to decisions related to the management of human resources for health at local levels, including hiring and firing of personnel, among others.

In your current (or previous) work, could you please tell me more about examples of decisions that you are making within the scope of human resources for health management?<br>*Probe: Are you able to*<br>• *Change the number and types of HRH needed to be hired at the local level?*<br>• *Create new job positions to meet the needs for HRH at the local level?*<br>• *Recruit or hire the person you want for the HRH position?*<br>• *Supervise or have authority over your local HRH?*<br>• *Evaluate the performance of your local HRH?*<br>• *Promote, discipline, transfer, replace, or fire (and the like) the local HRH when necessary?*<br>• *Provide incentives to recognize good work of local HRH?*<br>• *Others?*
29. In each of the examples that you described, would you say that you are able to make these decisions comfortably well? Or are you having difficulties in making such decisions <u>and</u> in executing those decisions? Please explain further.<br>*Probe: What usually are your considerations (or what do you look at) that lead to your decisions here?*<br>*Probe: Is your actual decision the desired one? Why or why not?*<br>*Probe: Ask informant to classify his/her decision space for this function into none, narrow, moderate, or wide.*<br>*Probe: What changes in decision space, if any, are necessary for this function? And what changes in the conditions are needed to effect such changes?*<br>*Probe: How do you assess if the decisions you made were right or wrong?*<br>*Probe: Do you make an attempt to compare or share experiences with other regions/provinces/LGUs etc.?*
Accountability
30. In your opinion, do you think that the decisions you are making related to HRH management are considered <u>accountable</u>? Please explain further.<br>*Probe: If not accountable, ask informant to elaborate on how to make decision-making more accountable.*
31. In your opinion, do you think that the decisions related to HRH management are considered <u>transparent</u>? Please explain further.<br>*Probe: Can the public easily access information related to HRH management?*<br>*Probe: If not transparent, ask informant to elaborate on how to make decision-making more transparent.*
32. Are you able to ensure <u>an inclusive approach</u> to decision-making? How are <u>stakeholders able to participate</u> in HRH management?<br>*Probe: At the end of the day, is your decision an individual decision or a collective decision?*<br>*Probe: If an inclusive approach is not adequate, ask the informant to elaborate on how to make it adequate.*

33. In your opinion, who are the <u>(3) three most important actors or stakeholders</u> who should be making decisions regarding HRH management at local levels? Please explain why.<br>*Probe: Which institution should take the lead in coordinating such decision-making?*
Capacity
34. In your personal assessment, would you say that you have an adequate capacity for making decisions related to HRH management? Please explain further.
35. What do you think could be strategies to build capacity for decision-making in HRH management in the Philippines?<br>*Probe: Who or which agency should be implementing the capacity building strategies suggested?*
**Service organization or delivery**
Decision Space
36. Simply put, service organization or delivery refers to decisions related to the implementation of health programs and services at local levels. In your current (or previous) work, could you please tell me more about examples of decisions that you are making within the scope of service organization or delivery?<br>*Probe: Are you able to*<br>• *Provide local health programs or public health measures that are new or different from those already being provided or those recommended by the national government?*<br>• *Decide which types of health facilities are needed in the locality? And when to build, renovate, or close (and the like) local health facilities?*<br>• *Decide which supplies (e.g. drugs, equipment, etc.) to buy, at which quantities, and by which mechanism to buy these?*<br>• *Engage the private sector in the delivery of some local health services?*<br>• *Others?*
37. In each of the examples that you described, would you say that you are able to make these decisions comfortably well? Or are you having difficulties in making such decisions <u>and</u> in executing those decisions? Please explain further.<br>*Probe: What usually are your considerations (or what do you look at) that lead to your decisions here?*<br>*Probe: Is your actual decision the desired one? Why or why not?*<br>*Probe: Ask informant to classify his/her decision space for this function into none, narrow, moderate, or wide.*<br>*Probe: What changes in decision space, if any, are necessary for this function? And what changes in the conditions are needed to effect such changes?*<br>*Probe: How do you assess if the decisions you made were right or wrong?*<br>*Probe: Do you make an attempt to compare or share experiences with other regions/provinces/LGUs etc.?*

Accountability
38. In your opinion, do you think that the decisions you are making related to service organization and delivery are considered <u>accountable</u>? Please explain further.<br>*Probe: If not accountable, ask informant to elaborate on how to make decision-making more accountable.*
39. In your opinion, do you think that the decisions related to service organization and delivery are considered <u>transparent</u>? Please explain further.<br>*Probe: Can the public easily access information related to service organization and delivery?*<br>*Probe: If not transparent, ask informant to elaborate on how to make decision-making more transparent.*
40. Are you able to ensure <u>an inclusive approach</u> to decision-making? How are <u>stakeholders able to participate</u> in service organization and delivery?<br>*Probe: At the end of the day, is your decision an individual decision or a collective decision?*<br>*Probe: If an inclusive approach is not adequate, ask the informant to elaborate on how to make it adequate.*
41. In your opinion, who are the <u>(3) three most important actors or stakeholders</u> who should be making decisions regarding service organization and delivery at local levels? Please explain why.<br>*Probe: Which institution should take the lead in coordinating such decision-making?*
Capacity
42. In your personal assessment, would you say that you have an adequate capacity for making decisions related to service organization and delivery? Please explain further.
43. What do you think could be strategies to build capacity for decision-making in HRH management in the Philippines?<br>*Probe: Who or which agency should be implementing the capacity building strategies suggested?*
**Other dimensions that help improve the implementation of devolution**
44. In your opinion, aside from decision-making, accountability, and capacity, what else must be considered, if any, to improve health system performance in the context of devolution?
45. ***Is there anything more that you would like to add or do you have any other comments about devolution in the Philippines which we have not yet discussed?***
Time finished:

**Closing**
• Stop the audio recording<br>• Describe next steps<br>• Say thank you<br>• Give a token of appreciation
